# The oncogenic role of treacle ribosome biogenesis factor 1 (*TCOF1*) in human tumors: a pan-cancer analysis

**DOI:** 10.18632/aging.203852

**Published:** 2022-01-30

**Authors:** Wei Gu, Le Sun, Jian Wang, Xiaowei Chen

**Affiliations:** 1 Department of Otolaryngology Head and Neck, Peking Union Medical College Hospital, Chinese Academy of Medical Sciences and Peking Union Medical College, Beijing 100730, China

**Keywords:** *TCOF1*, pan-cancer, bioinformatics, biomarker, prognosis, tumor microenvironment

## Abstract

Treacle ribosome biogenesis factor 1 (*TCOF1*) plays a crucial role in multiple processes, including ribosome biogenesis, DNA damage response (DDR), mitotic regulation, and telomere integrity. However, its role in cancers remains unclear. We aimed to visualize the expression, prognostic, and mutational landscapes of *TCOF1* across cancers and to explore its association with immune infiltration. In this work, we integrated information from TCGA and GEO to explore the differential expression and prognostic value of *TCOF1*. Then, the mutational profiles of *TCOF1* in cancers were investigated. We further determined the correlation between *TCOF1* and immune cell infiltration levels. Additionally, we determined correlations among certain immune checkpoints, microsatellite instability, tumor mutational burden (TMB), and *TCOF1*. Potential pathways of *TCOF1* in tumorigenesis were analyzed as well. In general, tumor tissue had a higher expression level of *TCOF1* than normal tissue. The prognostic value of *TCOF1* was multifaceted, depending on type of cancer. *TCOF1* was correlated with tumor purity, CD8+ T cells, CD4+ T cells, B cells, neutrophils, macrophages, and dendritic cells (DCs) in 6, 14, 16, 12, 20, 13, and 17 cancer types, respectively. *TCOF1* might act on ATPase activity, microtubule binding, tubulin binding, and catalytic activity (on DNA), and participate in tumorigenesis through “cell cycle” and “cellular-senescence” pathways. *TCOF1* could affect pan-cancer prognosis and was correlated with immune cell infiltration. “Cell cycle” and “cellular-senescence” pathways were involved in the functional mechanisms of *TCOF1*, a finding that awaits further experimental validation.

## INTRODUCTION

The treacle ribosome biogenesis factor 1 (*TCOF1*) gene is located on the long arm of chromosome 5 at the 5q32-33.3 locus and encodes treacle phosphoprotein [1, 2]. *TCOF1* was initially found as a gene related to Treacher Collins syndrome (TCS), a rare genetic disorder characterized by severe craniofacial defects, external ear deformation, and hearing impairment [3, 4]. The role of *TCOF1* in TCS has been extensively studied [5–7], and our previous works have identified novel *TCOF1* mutations in TCS [8, 9]. Recently, *TCOF1* has been reported to play crucial roles in multiple processes, including ribosome biogenesis [10], deoxyribonucleic acid (DNA) damage response (DDR) [11, 12], mitotic regulation [13], and telomere integrity [14, 15]. However, little is known about its role in carcinogenesis. Given that *TCOF1* participates in several key cellular processes, in this study we aimed to investigate the part it plays in human cancers.

Cancer remains a major public-health problem worldwide and has been a leading cause of death in the past several decades [16, 17]. Emerging therapies that include chemotherapy, radiotherapy, and immune checkpoint blockade targeting programmed death-1 (*PD-1*) and cytotoxic T-lymphocyte–associated protein 4 (*CTLA-4*) have shown great success in the treatment of several cancer types [18–20]. However, a considerable number of patients benefit little from available immunotherapies, and their prognoses remain poor. This dilemma emphasizes the importance of deeply understanding the mechanism underlying tumorigenesis. With the emergence of large-scale, multi-omics, and publicly accessible databases containing sample data from different types of cancer, it is now possible to analyze and evaluate the role of certain genes of interest, namely *TCOF1* in this study, at the pan-cancer level.

In this work, we visualized the expression and prognostic landscapes of *TCOF1* in pan-cancer based on published data. In addition, mutations to *TCOF1* and the gene’s relationships with certain genomic signatures were also explored. We further evaluated *TCOF1*’s relationships with immune cell infiltration, immune checkpoints, and immunotherapy responses. Finally, potential pathways involving *TCOF1* in tumor pathogenesis were also investigated.

## RESULTS

### Expression level of *TCOF1* in various cancers

**Figure 1:**
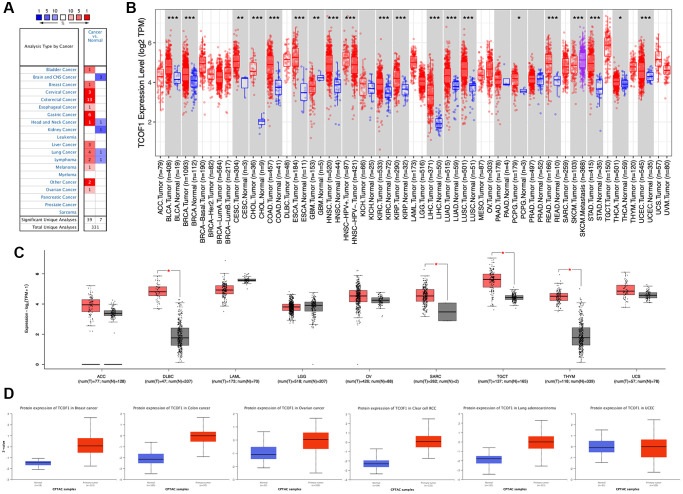
***TCOF1* expression levels in cancers.** (**A**) Differential-expression data for *TCOF1* in various types of cancer, compared with corresponding normal tissues, in Oncomine. (**B**) *TCOF1* expression levels in different tumor types from TCGA were analyzed using TIMER2. (**C**) Comparisons of *TCOF1* expression levels between tumor tissues from TCGA and normal tissues from GTEx. (**D**) *TCOF1* protein (treacle) expression levels in BC, CC, OC, CCRCC, LUAD, and UCEC. ^*^P < 0.05; ^**^P < 0.01; ^***^P < 0.001.

We examined *TCOF1* mRNA expression levels in various cancer types by analyzing TCGA data via Oncomine. The results showed that *TCOF1* expression in tumors was significantly higher than in normal tissues in many cancers, including bladder, breast, cervical, colorectal, esophageal, gastric, head and neck, liver, lung, and ovarian, as well as in melanoma and lymphoma. However, in certain studies, *TCOF1* was less expressed in brain, central nervous system (CNS), head and neck, kidney, and lung cancers and in lymphoma (Figure 1A).

To further evaluate differential expression of *TCOF1* in pan-cancer, we compared RNA sequencing data from TCGA using TIMER. As shown in Figure 1B, *TCOF1* expression was significantly higher in bladder urothelial carcinoma (BLCA), breast invasive carcinoma (BRCA), cervical squamous-cell carcinoma (CESC), endocervical adenocarcinoma (ECA), cholangiocarcinoma (CHOL), colon adenocarcinoma (COAD), esophageal carcinoma (ESCA), head and neck squamous-cell carcinoma (HNSC), clear-cell renal-cell carcinoma (CCRCC), papillary renal-cell carcinoma (PRCC), liver hepatocellular carcinoma (LIHC), lung adenocarcinoma (LUAD), lung squamous-cell carcinoma (LUSC), pheochromocytoma and paraganglioma (PCPG), rectal adenocarcinoma (READ), stomach adenocarcinoma (STAD), thyroid carcinoma (THCA), and uterine corpus endometrial carcinoma (UCEC). Notably, *TCOF1* expression in skin cutaneous melanoma (SKCM) metastatic tissue was remarkably higher than in respective primary tumor tissue. Lower expression of *TCOF1* in tumor was found only in glioblastoma multiforme (GBM). For certain tumors lacking normal-tissue data in TCGA, we used corresponding normal tissues from the GTEx dataset as controls and compared differences in *TCOF1* expression using GEPIA2. As shown in Figure 1C, expression of *TCOF1* was upregulated in diffuse large B-cell lymphoma (DLBCL), sarcoma (SARC), testicular germ cell tumor (TGCT), and thymoma (THYM). However, we did not see significant differences in other tumors, including adrenocortical carcinoma (ACC), acute myeloid leukemia (LAML), brain lower-grade glioma (LGG), ovarian serous cystadenocarcinoma (OV), and uterine carcinosarcoma (UCS).

Based on the CPTAC dataset, we then evaluated protein expression of *TCOF1* in pan-cancer via the UALCAN portal. Compared with normal tissues, expression of *TCOF1* total protein was higher in breast cancer (BC), colon cancer (CC), ovarian cancer (OC), CCRCC, and LUAD, but not in UCEC (Figure 1D). We also investigated *TCOF1* protein expression in 20 types of cancer using the HPA cohort. IHC staining results showed that high expression of *TCOF1* could be observed in colorectal (36.4%), testicular (16.67%), pancreatic (11.11%), urothelial (10%), stomach (9.09%), liver (8.33%), endometrial (8.33), ovarian (8.33%), renal (8.33%), and skin cancers (8.33%; Supplementary Figure 1).

### *TCOF1* expression and cancer patients’ prognoses

**Figure 2:**
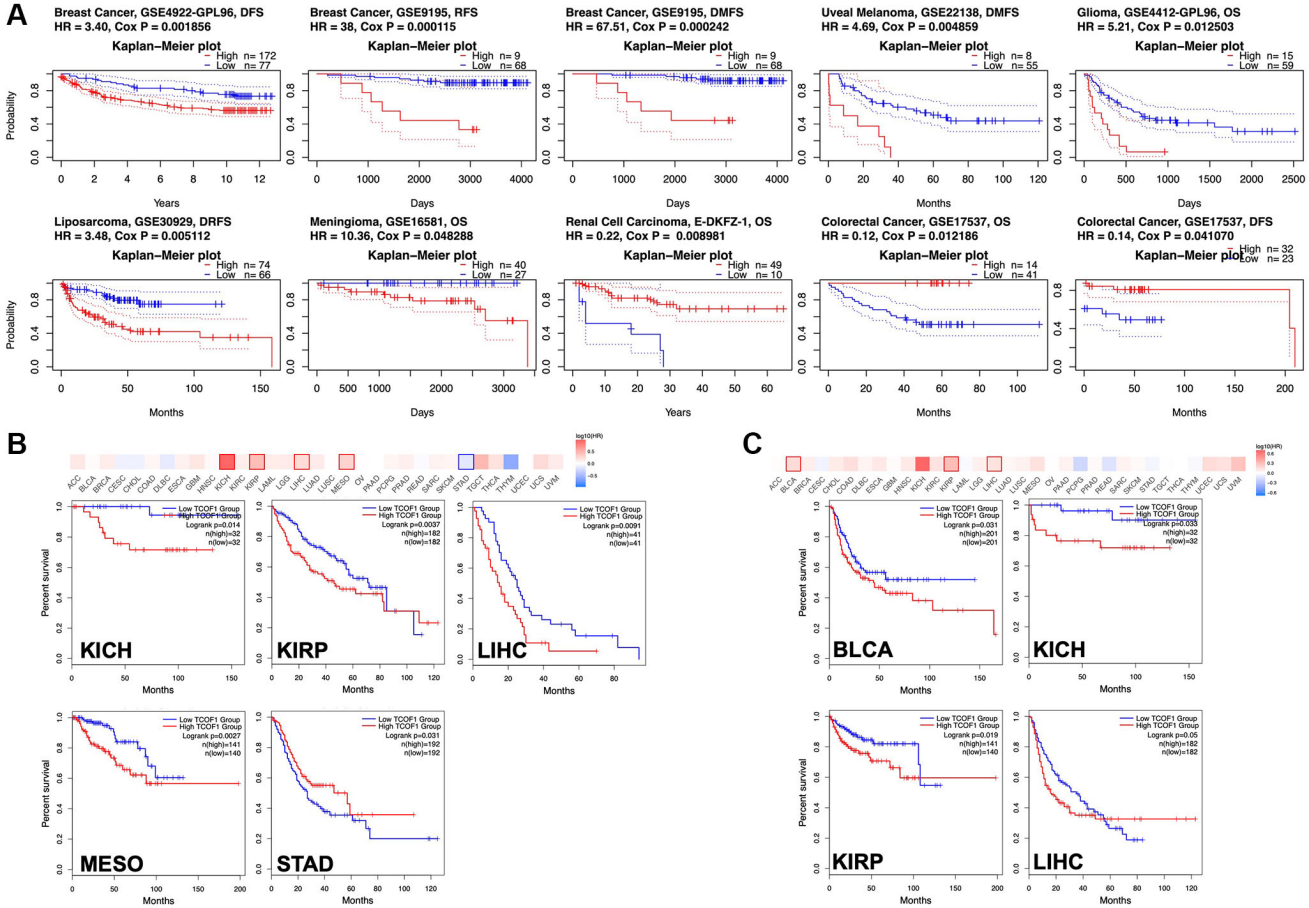
**Survival analysis based on expression level of *TCOF1*.** (**A**) Survival curves with significance in eight cohorts from the GEO dataset (GSE4922-GPL96, GSE9195, GSE22138, GSE4412-GPL96, GSE30929, GSE16581, E-DKFZ-1, and 17537). (**B**, **C**) Survival heatmap and Kaplan–Meier curves with positive results from (**B**) OS and (**C**) DFS analyses of different tumors in TCGA based on *TCOF1* expression.

To understand how *TCOF1* affects the prognoses of cancer patients, we analyzed the relationship between survival outcomes and *TCOF1* expression levels via PrognoScan. *TCOF1* expression was significantly correlated with prognosis in seven cancer types: BC, uveal melanoma (UVM), liposarcoma, renal-cell carcinoma (RCC), glioma, meningioma, and colorectal cancer (CRC; Figure 2A). Of these, high expression levels of *TCOF1* were detrimental to patient prognosis in BC, UVM, liposarcoma, glioma, and meningioma, but they played a protective role in RCC and CC. Detailed data are shown in a forest plot (Supplementary Figure 2).

We further investigated the association between *TCOF1* expression and cancer patients’ prognoses in TCGA databases via GEPIA2. As shown in Figure 2B, high expression levels of *TCOF1* were related to poor prognosis for overall survival (OS) in kidney chromophobe (KICH; *P* = 0.014), PRCC (*P* = 0.0027), LIHC (*P* = 0.0037), and mesothelioma (MESO, *P* = 0.0091) in TCGA datasets. Disease-free survival (DFS) analysis results, shown in Figure 2C, revealed that high expression of *TCOF1* was correlated with poor prognosis in BLCA (*P* = 0.031), KICH (*P* = 0.033), PRCC (*P* = 0.019), and LIHC (*P* = 0.05) in TCGA datasets. However, lower expression levels of *TCOF1* were linked to poor OS for STAD (*P* = 0.031, Figure 2A).

### Mutation profiles and genome-wide association of *TCOF1*

**Figure 3:**
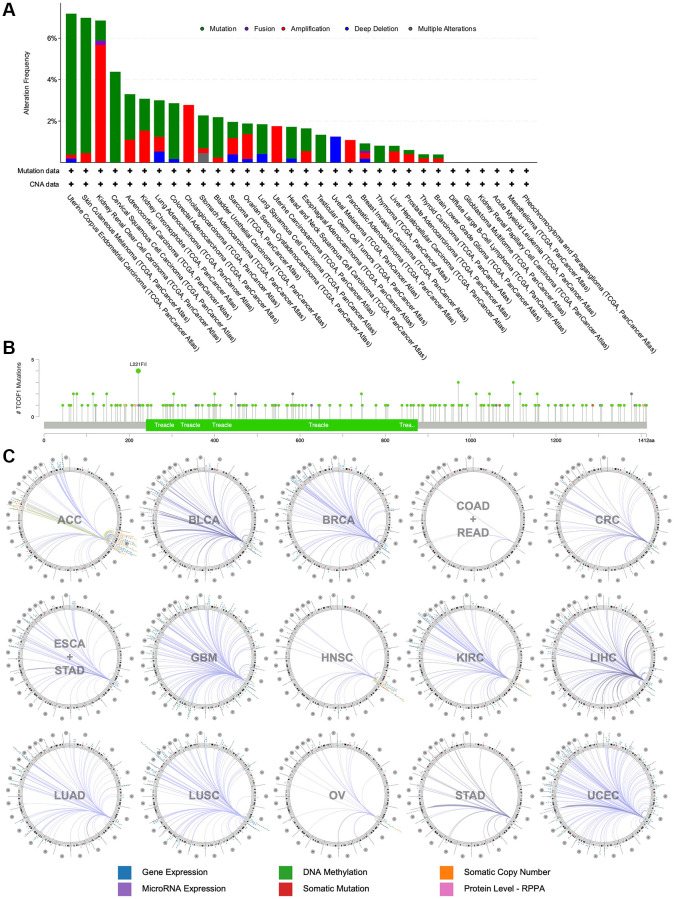
**Mutational landscape and genome-wide association of *TCOF1*.** (**A**) Alternation types and frequency of *TCOF1* in different types of cancer. (**B**) Mutation sites of *TCOF1* across cancers. (**C**) The genome-wide correlation between *TCOF1* and other signatures from TCGA, visualized using Regulome Explorer.

Using cBioPortal, we investigated the mutation frequency of *TCOF1* in 10,967 samples from 32 TCGA studies. As shown in Figure 3A, the highest alteration frequency of *TCOF1* (>6%) appeared in UCEC patients, with “mutation” as the primary type. The “amplification” type of copy number alteration (CAN) was the primary type in CCRCC cases, showing an alteration frequency of >5%. Notably, all UVM cases with genetic alterations (~2% frequency) had copy number deletion of *TCOF1* (Figure 3A). We detected 196 mutations (including 164 missense, 22 truncating, 2 inframe, 4 fusion, and 2 duplicate mutations in patients with multiple samples) and located their sites between amino acids 0 and 1412. Of these, L221F/I (from 4 UCEC samples) was the most frequent mutation site (Figure 3B). The details of all mutation profiles are summarized in Supplementary Table 1.

We then used the Regulome Explorer to further inspect the genome-wide association between *TCOF1* and certain genomic signatures. Expression of *TCOF1* and its correlations with other variables in cancers on the chromosomal level (including DNA methylation, somatic copy number, microRNA expression, somatic mutation, and protein level) is displayed in Circos plots (Figure 3C). Based on data from TCGA, associations could be detected between *TCOF1* and other signatures in ACC, BLCA, BRCA, COAD, READ, CRC, ESCA, STAD, GBM, HNSC, CCRCC, LIHC, LUAD, LUSC, OV, STAD, and UCEC within the context of genomic coordinates. Detailed data are listed in Supplementary Table 2.

We next examined the relationship between *TCOF1* expression and expression levels of four DNA-methyltransferases (DNMTs) in pan-cancer. As shown in Supplementary Figure 3, we observed a significant and positive correlation between *TCOF1* and DNMTs in almost all types of cancer except UCS and CHOL. This indicated that upregulated expression of *TCOF1* in different cancers might contribute to DNA methylation.

### Relationship between *TCOF1* expression and immune cell infiltration

**Figure 4:**
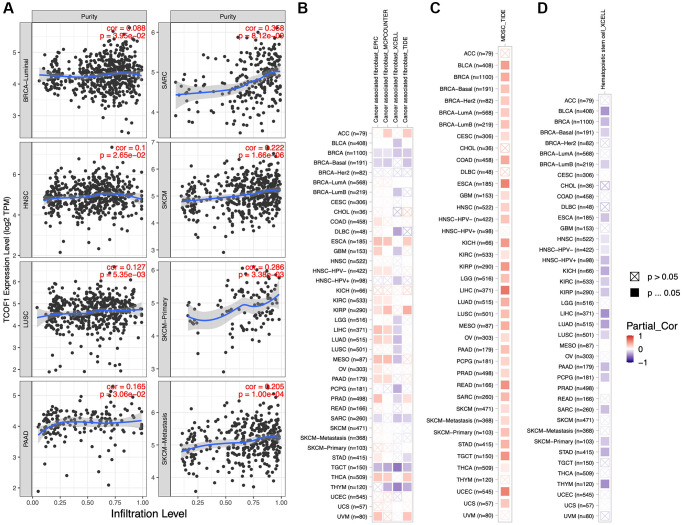
**Associations of *TCOF1* expression with tumor purity and immune infiltration.** (**A**) *TCOF1* expression was positively correlated with tumor purity in BRCA-Luminal, HNSC, LUSC, pancreatic adenocarcinoma (PAAD), SARC, and SKCM based on TCGA data. (**B**–**D**) Correlation between *TCOF1* expression level and infiltration levels of (**B**) CAFs, (**C**) MDSCs, and (**D**) HSCs across all types of cancer in TCGA.

We further investigated the correlation between infiltration levels of different immune cells in pan-cancer and *TCOF1* expression level. The results indicated that high *TCOF1* expression was positively related to tumor purity in six types of cancer (Figure 4A). Additionally, *TCOF1* expression level was significantly associated with infiltration levels of Cluster of Differentiation 8–positive (CD8+) T cells, CD4+ T cells, B cells, neutrophils, macrophages, and dendritic cells (DCs) in 14, 16, 12, 20, 13, and 17 cancer types, respectively (Supplementary Figure 4). Cancer-associated fibroblasts (CAFs) are among the most abundant stromal components of the tumor microenvironment (TME), and they can build up and remodel the extracellular-matrix (ECM) structure to facilitate tumor invasion [21]. We observed a significantly positive correlation of *TCOF1* expression and CAF infiltration level in ESCA, PRCC, and THCA, but noted a negative correlation in BRCA, TGCT, and THYM (Figure 4B). In addition, abundance of myeloid- derived suppressor cells (MDSCs), which can inhibit T-cell function and thus contribute to the pathogeneses of various diseases, was found to be positively correlated with *TCOF1* in almost all cancer types (Figure 4C). Finally, hematopoietic stem cells (HSCs), had a negative relationship with *TCOF1* expression in most types of cancer (Figure 4D).

### Association between *TCOF1* expression and immunotherapy

**Figure 5:**
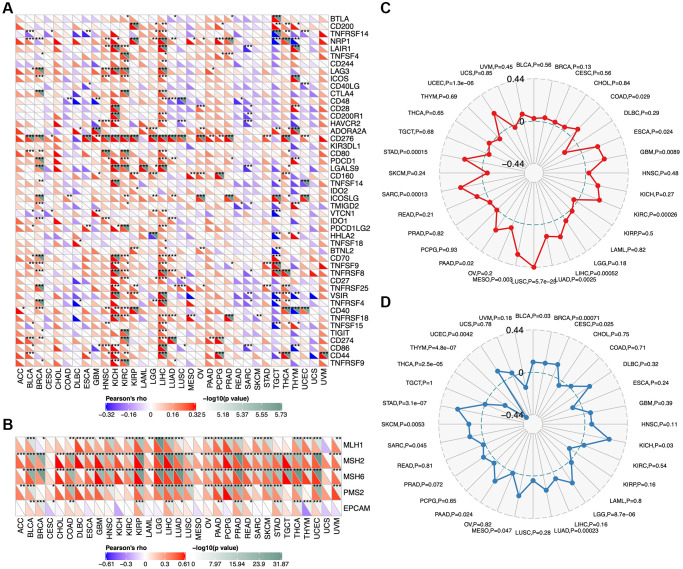
**Relationship of *TCOF1* and immunotherapy.** (**A**) Correlations between *TCOF1* and confirmed immune checkpoints across cancers. (**B**) Correlations between *TCOF1* and five essential genes for MMR in multiple cancers. (**C**, **D**) Correlations of *TCOF1* with (**C**) MSI and (**D**) TBM. ^*^P < 0.05; ^**^P < 0.01; ^***^P < 0.001.

We analyzed correlations between *TCOF1* and certain immune checkpoints, including B- and T-lymphocyte attenuator (*BTLA*), leukocyte-associated immunoglobulin-like receptor 1 (*LAIR1*), CD244, lymphocyte activation gene 3 (*LAG3*), inducible T-cell costimulator (ICOS), CD40 ligand (CD40LG), *CTLA4*, CD48, CD28, CD200 receptor 1 (CD200R1), CD80, programmed cell death protein 1 (*PDCD1*), transmembrane and immunoglobulin domain containing 2 (*TMIGD2*), programmed cell death 1 ligand 2 (*PDCD1LG2*), CD27, *TIGIT*, CD86, and tumor necrosis factor receptor superfamily 9 (TNFRSF9). As shown in Figure 5A, *TCOF1* had significant correlations with most of the immune checkpoints in KICH, CCRCC, and LIHC. Notably, in 20 of 33 types of cancer, CD276 expression was remarkably associated with *TCOF1*. Based on these results, we hypothesized a synergistic effect of *TCOF1* and immune checkpoints in cancers. However, based on the data from TISIDB, we did not observe any significant difference in *TCOF1* expression level between immunotherapy responders and non-responders (Supplementary Table 3).

Microsatellites are short tandem repeat (STR) DNA sequences distributed throughout the human genome and prone to replication errors, which can normally be repaired by the mismatch repair (MMR) system [22]. The MMR system is usually dependent on four key genes: mutL homologue 1 (*MLH1*), postmeiotic segregation increased 2 (*PMS2*), mutS homologue 2 (*MSH2*), and mutS 6 (*MSH6*). The epithelial-cell adhesion molecule (*EPCAM*) gene has also been identified as a key MMR gene [23]. We analyzed the correlation between *TCOF1* expression and MSI, a strong mutator phenotype with a deficient MMR system, in different types of cancers and found that *TCOF1* was significantly and positively correlated with *MLH1*, *MSH2*, *MSH6*, and *PMS2* in almost all types but negatively correlated with *EPCAM* in CESC, KICH, and THYM (Figure 5B). In addition, high-MSI tumors appeared to express higher levels of *TCOF1* than genetically stable ones in CCRCC, LIHC, LUSC, SARC, STAD, and UCEC (*P* < 0.001; Figure 5C). TMB, the total number of mutations per coding area of a tumor genome, is a biomarker of sensitivity to ICIs. We analyzed the correlation between *TCOF1* expression and TMB across various cancer types. The results showed that *TCOF1* expression was positively correlated with TMB in BRCA, LGG, LUAD, and STAD (*P* < 0.001) but negatively correlated with TMB in THCA and THYM cohorts (*P* < 0.001; Figure 5D). However, all significant correlations coefficients of *TCOF1* with MSI or TBM were <0.5, which is insufficient to predict a cancer patient’s response to immunotherapy (Supplementary Table 3).

### *TCOF1*-related genes and potential pathways in cancer

**Figure 6:**
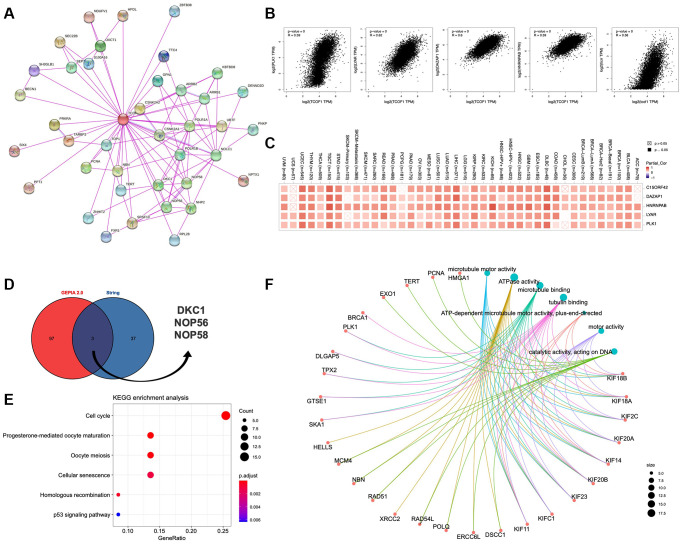
**Pathway enrichment analysis of *TCOF1*.** (**A**) Available experimentally determined *TCOF1*-binding proteins discovered using the STRING tool. (**B**) Correlation of *TCOF1* expression with that of the top 5 related genes via GEPIA2 (*DAZAP1*, *HNRNPAB*, *LYAR*, *TICRR/C15orf42*, and *PLK1*). (**C**) The corresponding heatmap data of these top 5 genes in detailed cancer types. (**D**) *DKC1*, *NOP56*, and *NOP58* are three common genes (proteins) of STRING-based *TCOF1*-binding proteins and GEPIA2-based *TCOF1*-correlated genes. (**E**) KEGG pathway analysis based on *TCOF1*-binding and -interacting genes. (**F**) The cnetplot of Molecular Function data in GO analysis.

To further understand the mechanism of *TCOF1* in tumor pathogenesis, we investigated genes and proteins related to *TCOF1* expression and conducted pathway enrichment analyses thereof. Using STRING, we identified *TCOF1*-binding proteins supported by experimentally available evidence. The interaction network of these proteins and *TCOF1* is shown in Figure 6A. Then, we used GEPIA2 to determine the top 100 genes correlated with *TCOF1* expression in tumor data from TCGA projects. As shown in Figure 6B, *TCOF1* expression was most positively significantly correlated to expression of deleted in azoospermia-associated protein 1 (*DAZAP1*), heterogeneous nuclear ribonucleoprotein A/B (*HNRNPAB*), Ly1 antibody reactive (*LYAR*), DNA topoisomerase II binding protein 1 (*TOPBP1*), interacting checkpoint and replication regulator (*TICRR*, also known as *C15orf42*), and polo-like kinase 1 (*PLK1*). The corresponding detailed heatmap data of the various cancer types is displayed in Figure 6C. Intersection analysis of STRING-based *TCOF1*-binding proteins and GEPIA2-based *TCOF1*-correlated genes showed three common members: dyskerin pseudouridine synthase 1 (DKC1), nucleolar protein 56 (*NOP56*), and *NOP58* (Figure 6D).

Next, we used the identified genes to perform KEGG and GO enrichment analyses. KEGG results (Figure 6E) indicated that *TCOF1* might influence tumorigenesis through “cell cycle” and “cellular-senescence” pathways. GO Molecular Function analysis suggested most of the *TCOF1*-related genes were associated with adenosine triphosphatase (ATPase) activity, microtubule binding, tubulin binding, catalytic activity (acting on DNA), and other functions (Figure 6F).

## DISCUSSION

The *TCOF1* gene, located on the long arm of chromosome 5 at the 5q32-33.3 locus, is composed of 28 exons [2, 24]. In transcription, *TCOF1* is alternatively transcribed and spliced into several mRNA isoforms. Most of these isoforms contain exon 6A/treacle, a translational product of exon 6A– contained *TCOF1* mRNA isoform [24], which is a nucleolar phosphoprotein with 1488 amino acids and a low-complexity three-domain structure [25]. *TCOF1* and treacle are reported to regulate multiple key cellular processes, including ribosome biogenesis, mitosis, proliferation, cellular response to DNA damage, and apoptosis [26]. While *TCOF1*-related mechanisms in TCS have been extensively studied, research focused on the role of *TCOF1* in malignancies is limited.

In this work, we used integrated bioinformatics methods to comprehensively analyze the role of *TCOF1* and its corresponding protein treacle in different types of cancers. According to the results of expression analysis, *TCOF1* was upregulated in most cancer types when compared to corresponding normal tissues (Figure 1A–1C). The expression level of treacle protein was significantly higher in BC, CC, OC, CCRCC, and LUAD, but not in UCEC (Figure 1D). As subsequently confirmed in mutation profiles of *TCOF1*, UCEC patients had the highest alternation frequency, with “mutation” as the primary type (Figure 3A). Survival analysis results then indicated that *TCOF1* was correlated with prognosis in several cancers, including BC, UVM, liposarcoma, RCC, glioma, meningioma, and CRC (Figure 2). Multifaceted, even contradictory, prognostic values of *TCOF1* in different types of cancer might be attributable to distinct underlying mechanisms in certain tumors; heterogeneous data collection approaches; and other clinical factors such as gender, ethnicity, and tumor stage. These results indicated the potential prognostic value of *TCOF1* in different types of cancer.

By using different algorithms, we found significant correlations between *TCOF1* and infiltration levels of several types of immune cells, including CAFs and MDSCs (Figure 5). Immune cells interact with tumor cells in the TME and therefore play vital roles in anti- or pro-tumor incidents. For example, CAFs, the prominent components of stromal cells, are reportedly associated with poor outcomes, therapy resistance, and tumor recurrence in various types of cancer [27]. Our findings indicated that *TCOF1* might exert an essential effect on cancer development and it might serve as a potential therapy target. However, a cause–effect relationship could not be established in this study. Additionally, pathway enrichment analyses showed that TCOF1 might act mainly on ATPase activity, microtubule binding, tubulin binding, and catalytic activity (on DNA) and participate in tumorigenesis through “cell cycle” and “cellular-senescence” pathways (Figure 6). This finding was partially in line with those of existing *TCOF1*-related studies, as further discussed below.

Treacle acts as a key regulator in the biogenesis of ribosomes, one of the most important cell processes and essential for nearly 95% of total transcription [28]. Ribosome biogenesis has three main stages: transcription of ribosomal DNA (rDNA) into precursor ribosomal RNA (pre-rRNA), post-transcriptional processing from pre-rRNA to mature rRNA, and ribosome assembly [28, 29]. The first stage starts with the formation of the pre-initiation complex around the rDNA promoter region in the nucleolus. The latter consists of upstream binding factor (*UBF*), transcription initiation factor 1 alpha (*TIF1-α*), selectivity factor 1 (*SL1*, also known as *TIF1-β*), and DNA polymerase I (*Pol I*) [30, 31]. Treacle plays a crucial role in ribosome biogenesis in that its different domains can bind and recruit *UBF*, *Pol I*, and *Nopp140* to the rDNA promoter [10]. In addition, treacle is an activator of *UBF*, an important regulator in the transcription of rDNA [32]. Insufficient treacle leads to the dispersion and dysfunction of *UBF* and *Pol I* and the resulting inhibition of rRNA transcription [11, 32]. During the second stage, treacle interacts with ribonucleoprotein *NOP56* (Figure 6D) to regulate post-transcriptional pre-rRNA modifications, including methylation; this ensures the flexibility of RNA strands, protects RNA from hydrolysis, and regulates translation in cells [33, 34]. In the cervical-carcinoma HeLa cell line, silencing of *TCOF1* leads to inhibition of rRNA transcription and attenuated cell proliferation [32]. It also causes dysfunction of an RNA helicase called DEAD-box RNA helicase 21 (*DDX21*) by relocating it from the nucleolus to the nucleoplasm [35]. *DDX21* has been reported to promote gastric-cancer (GC) proliferation and tumor growth [36].

Due to recombination between rDNA sequences from different chromosomes, the genes encoding rRNA are unstable and prone to damage [37]. DDR is a signal transduction pathway involving multiple repair mechanisms in cells, and it is closely associated with tumorigenesis [38, 39]. The DDR repair process for the reconstruction of double-stranded breaks is triggered by ataxia telangiectasia mutated (ATM) and ataxia telangiectasia and *Rad3*-related (ATR) kinases, and transcriptional silencing is then induced to save energy and to prevent collision between catalyzing complexes of transcription and repair [40]. Treacle plays an essential role in DDR mechanisms by recruiting nibrin (*NBS1*) and *TOPBP1*, the key adaptor proteins of ATM and ATR kinases, respectively [12, 37, 41]. Accumulation of *NBS1* mediated by treacle is crucial for stopping rRNA transcription in the DDR process, and *NBS1* overexpression is reportedly associated with chemoresistance and with tumor development and metastasis [11, 42–44]. Treacle can interact with *NBS1* and thereby contribute to cancer development. Furthermore, overexpression of *TCOF1* has been associated with radioresistance in acinar progenitor cells of rat salivary glands, but attenuated *TCOF1* expression can sensitize human osteosarcoma cells to irradiation [41, 45]. Since treacle recruits *TOPBP1* in the DDR process and the latter confers radioresistance in osteosarcoma [12], *TOPBP1* might mediate the effect of treacle on sensitivity to radiotherapy. However, the potential contribution of *TCOF1* to cancer progression and resistance to therapy needs to be elucidated in future studies.

Although we integrated information from different public databases to present a broad view of *TCOF1* in pan-cancer, our study had some limitations. First, we analyzed tumor tissue information mainly based on microarray and sequencing data, meaning that cellular-level analysis results might be systemically biased. Future studies using high-resolution methods, such as single-cell RNA sequencing [46], should be performed to overcome or minimize such bias. Second, via multiple databases, we conducted bioinformatic analyses of *TCOF1* expression and patient prognosis only, but *in vivo/vitro* experimental evidence on the cellular or molecular level is still needed to help clarify the role of *TCOF1* in tumors. Third, although we found significant correlations among immune cell infiltration levels, survival outcomes, potential pathways, and *TCOF1*, we failed to identify *TCOF1* as friend or foe in cancers due to conflicting results from different databases. Since tumorigenesis is a complex process involving multiple pathways, our study provided only preliminary findings on the oncogenic role of *TCOF1*; its exact role in certain type of cancers should be evaluated and validated more precisely and comprehensively.

In summary, we presented a broad view of *TCOF1*’s role in pan-cancer. *TCOF1* was upregulated in most types of cancers, and we believe it might serve as a prognostic biomarker depending on cancer type. Infiltration levels of several types of immune cells, including CAFs and MDSCs, were highly correlated with *TCOF1* expression, suggesting underlying mechanisms involving *TCOF1* and immunity in tumorigenesis that should be further explored. Future studies should focus on potential regulation of *TCOF1* by multiple oncogenic-signaling pathways.

## MATERIALS AND METHODS

### Databases

The Cancer Genome Atlas (TCGA; http://cancergenome.nih.gov) is a prestigious cancer genomics project funded by the National Cancer Institute (NCI; Bethesda, MD, USA), which has characterized >20,000 primary cancer and matched normal-tissue samples from various cancer types [47]. The Gene Expression Omnibus (GEO; https://www.ncbi.nlm.nih.gov/geo) is a publicly available genomics-data repository containing array- and sequence-based data [48]. The Genotype-Tissue Expression Project (GTEx; http://commonfund.nih.gov/GTEx) is a commonly funded data resource and tissue bank containing tissue-specific gene expression data [49]. The Clinical Proteomic Tumor Analysis Consortium (CPTAC; https://proteomics.cancer.gov/programs/cptac), also funded by the NCI, is a comprehensive database that aims to identify proteins in cancer genomes and related biological processes and that provides genomic and proteomic data from >1100 cancer patients [50]. Analyses in our study were conducted based on the data from TCGA, GEO, GTEx, and CPTAC.

### Expression analysis

Oncomine is a cancer microarray database and web-based data-mining platform aimed at facilitating discovery from genome-wide expression analyses [51]. In this study, we recorded differential-expression data of *TCOF1* between various cancer samples and corresponding normal tissues from Oncomine. Thresholds of *P*-values and fold change (FC) were 0.01 and 1.5, respectively. Next, we used the “Gene_DE” module of Tumor IMmune Estimation Resource 2 (TIMER2; http://timer.cistrome.org), an online tool for systematical analysis of immune infiltrates across diverse cancer types [52], to visualize expression differences of *TCOF1* in pan-cancer from TCGA data. For certain cancer types without normal-tissue data, we matched and compared them with corresponding normal-tissue data from GTEx via the “Expression DIY” panel of Gene Expression Profiling Interactive Analysis 2 (GEPIA2; http://gepia2.cancer-pku.cn), a portal for analyzing ribonucleic acid (RNA) sequencing expression data from the TCGA and GTEx projects [53]. Cutoffs of *P*-values and |Log2FC| were 0.01 and 1, respectively. *TCOF1* protein expression analysis in six cancer types based on CPTAC data was conducted on UALCAN (http://ualcan.path.uab.edu), a web portal for analyzing cancer omics data [54].

The Human Protein Atlas (HPA; https://www.proteinatlas.org/) is an online portal that contributes to the mapping of human proteins in tissues. We obtained protein expression levels and immunohistochemical (IHC) staining results of *TCOF1* protein in 20 types of cancer samples from HPA.

### Survival analysis based on *TCOF1*

PrognoScan http://dna00.bio.kyutech.ac.jp/PrognoScan/index.html is a publicly accessible and powerful platform for evaluating the association between a gene and clinical outcome in cancers [55]. We conducted survival analysis of different cancer types and explored its relationship with *TCOF1* expression on PrognoScan. We used the “forestplot” package in R studio (version 1.4.1103; R Foundation for Statistical Computing, Vienna, Austria) to summarize the results and drew a forest plot of them. In addition, given that PrognoScan is a collection of cancer microarray datasets, we then performed survival analysis based on TCGA data using the “Survival Analysis” panel of GEPIA2. The significance level was set to 0.05.

### *TCOF1*’s mutational landscape and correlation with genomic signatures

We employed cBioPortal (http://www.cbioportal.org), an open-access resource for interactive exploration of multidimensional cancer genomics datasets [56], to investigate the mutation profiles of *TCOF1* in different tissues. We chose data from 10,967 samples in 32 studies from TCGA and determined the frequency, types, and sites of *TCOF1* mutations in multiple kinds of cancer. Regulome Explorer (http://explorer.cancerregulome.org) is an online tool to search, filter, and visualize analytical results generated from TCGA data. We used this tool to explore the correlation between *TCOF1* expression and certain genomic signatures. The filter of associations was set as follows: −Log10(p) ≥2; Correlation ≥0.4; Max results = 200. We calculated the relationship between *TCOF1* expression and four DNA-methyltransferases (DNMTs) and presented the results as a circular plot using SangerBox (http://www.sangerbox.com/tool), a powerful computerized online tool for bioinformatics analysis.

### Immunity-related analysis

We used TIMER2 to explore the relationship between *TCOF1* expression and immune cell infiltration levels. The “Gene” module, as indicated on the TIMER2 website, allows users to select any gene of interest and visualize the correlation of its expression with immune infiltration levels in diverse cancer types. The association analysis is adjusted for tumor purity and calculated by multiple algorithms, including TIMER, xCell (https://xcell.ucsf.edu/), MCPcounter (https://github.com/ebecht/MCPcounter), CIBERSORT (https://cibersort.stanford.edu/), Epigenomics of Plants International Consortium (EPIC; https://www.plant-epigenome.org/), and quanTIseq (http://icbi.i-med.ac.at/software/quantiseq/doc/index.html). We analyzed the associations among microsatellite instability (MSI), tumor mutation burden (TMB), and *TCOF1* expression using SangerBox. Finally, we evaluated mutational and expression differences of *TCOF1* between immunotherapy responders and non-responders using the Tumor and Immune System Interaction Database (TISIDB; http://cis.hku.hk/TISIDB), a web portal for such interactions [57].

### *TCOF1*-related gene identification and functional-enrichment analysis

The Search Tool for the Retrieval of Interacting Genes/Proteins (STRING; https://string-db.org) is a database of functional protein association networks [58]. We used STRING to identify and visualize a *TCOF1*-binding protein network based on the experimental evidence, with a minimum interaction score of 0.15. In GEPIA2, the “Similar Genes Detection” pane was used to search for the top 100 genes similar to *TCOF1* in TCGA tumors. We computed the correlations between *TCOF1* and the top 5 similar genes in all types of cancers and presented them in scatter plots using the “Correlation Analysis” pane in GEPIA2. The results were also presented as a heatmap plot using the “Gene_Corr” module of TIMER2. Intersection analysis results of STRING and GEPIA2 results were displayed as a Venn diagram using the “VennDiagram” package in R studio. Next, we combined two sets of data to conduct Kyoto Encyclopedia of Genes and Genomes (KEGG) pathway analysis and Gene Ontology (GO) enrichment analysis. For both types of analyses, we used the “GO/KEGG clusterProfiler” module in Hiplot (https://hiplot.com.cn), a comprehensive web platform for visualizing scientific data. *P*- and *Q*-value thresholds were set at 0.01 and 0.05, respectively.

### Ethics statement

The studies involving human participants were reviewed and approved by all the research data based on the bioinformatic analysis of the open resources from the TIMER, Oncomine, TCGA, and GEPIA databases. Written informed consent for participation was not required for this study in accordance with the national legislation and the institutional requirements.

### Data availability statement

Publicly available datasets were analyzed in this study. The data can be found here: http://timer.cistrome.org, http://gepia2.cancer-pku.cn, http://ualcan.path.uab.edu, http://dna00.bio.kyutech.ac.jp/PrognoScan/index.html, http://www.cbioportal.org, https://hiplot.com.cn, https://string-db.org, http://cis.hku.hk/TISIDB, http://www.sangerbox.com/tool. However, the Oncomine database has been closed and it’s no longer available. The original contributions presented in the study are included in the Supplementary Material. Further inquiries can be directed to the corresponding author.

## Supplementary Materials

Supplementary Figures 1 and 3

Supplementary Figure 2

Supplementary Figure 4

Supplementary Table 1

Supplementary Table 2

Supplementary Table 3
