# Supplementary material for: The oncogenic role of treacle ribosome biogenesis factor 1 (TCOF1) in human tumors: a pan-cancer analysis
Source: Aging (Albany NY). 2022 Jan 30;14(2):943–60. doi: 10.18632/aging.203852 (PMC8833134; doi:10.18632/aging.203852)
Supplement: Supplementary Figures 1 and 3 [file aging-14-203852-s001.pdf]

SUPPLEMENTARY FIGURES

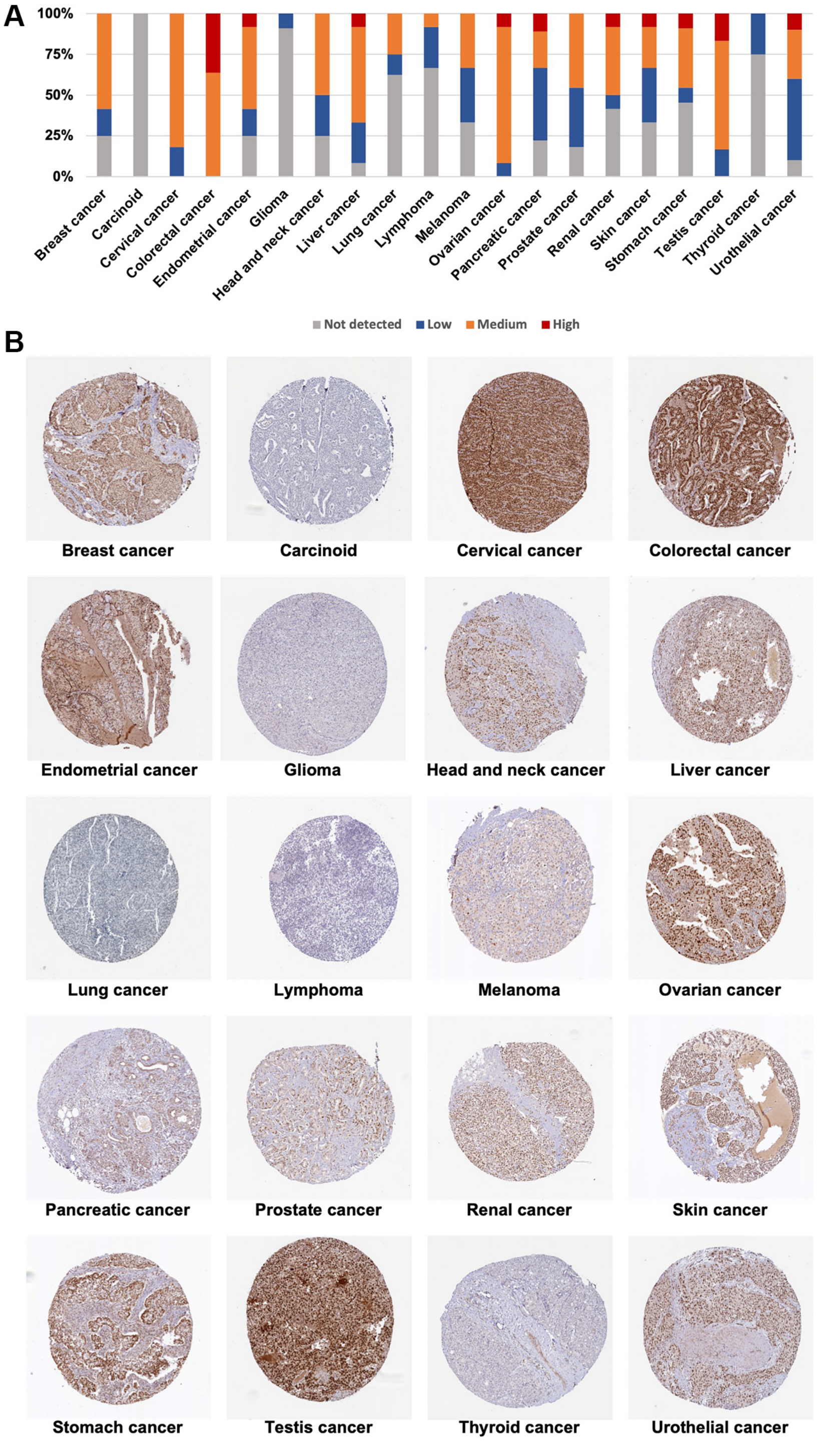

Supplementary Figure 1. (**A**) Chart of different expression levels of *TCOF1* protein in pan-cancer. (**B**) Representative IHC staining results of *TCOF1* protein in different cancer tissues.

Please browse Full Text version to see the data of Supplementary Figure 2.

Supplementary Figure 2. Forest plot of survival analyses of *TCOF1* in pan-cancer.

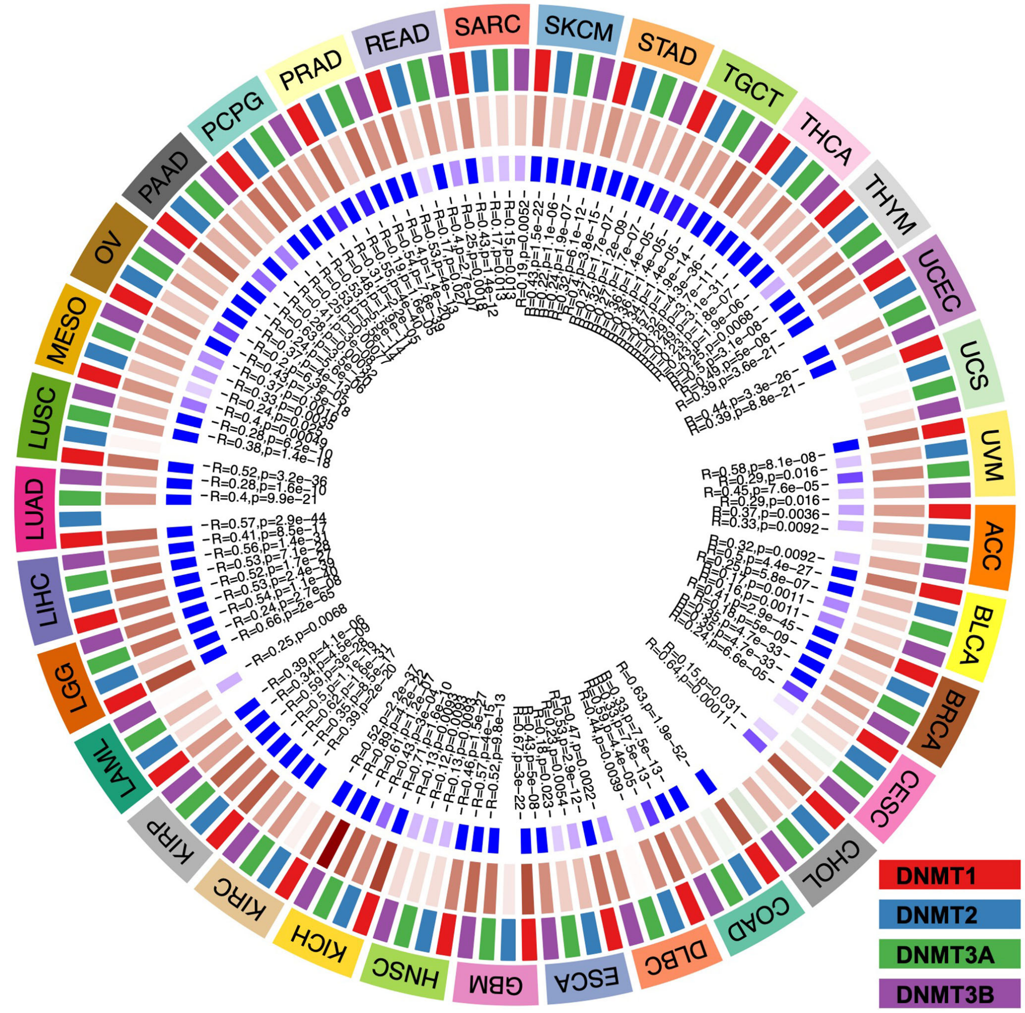

Supplementary Figure 3. Correlations of *TCOF1* expression with four DNA-methyltransferases.

Please browse Full Text version to see the data of Supplementary Figure 4.

Supplementary Figure 4. Correlation of *TCOF1* expression with infiltration levels of CD8^{+} T cells, CD4^{+} T cells, B cells, neutrophils, macrophages, and DCs.
