# Supplementary figures and images for: The oncogenic role of treacle ribosome biogenesis factor 1 (TCOF1) in human tumors: a pan-cancer analysis
Source: Aging (Albany NY). 2022 Jan 30;14(2):943–60. doi: 10.18632/aging.203852 (PMC8833134; doi:10.18632/aging.203852)

Survival Analyses of TCOF1 in Pan-cancer via Prognoscan

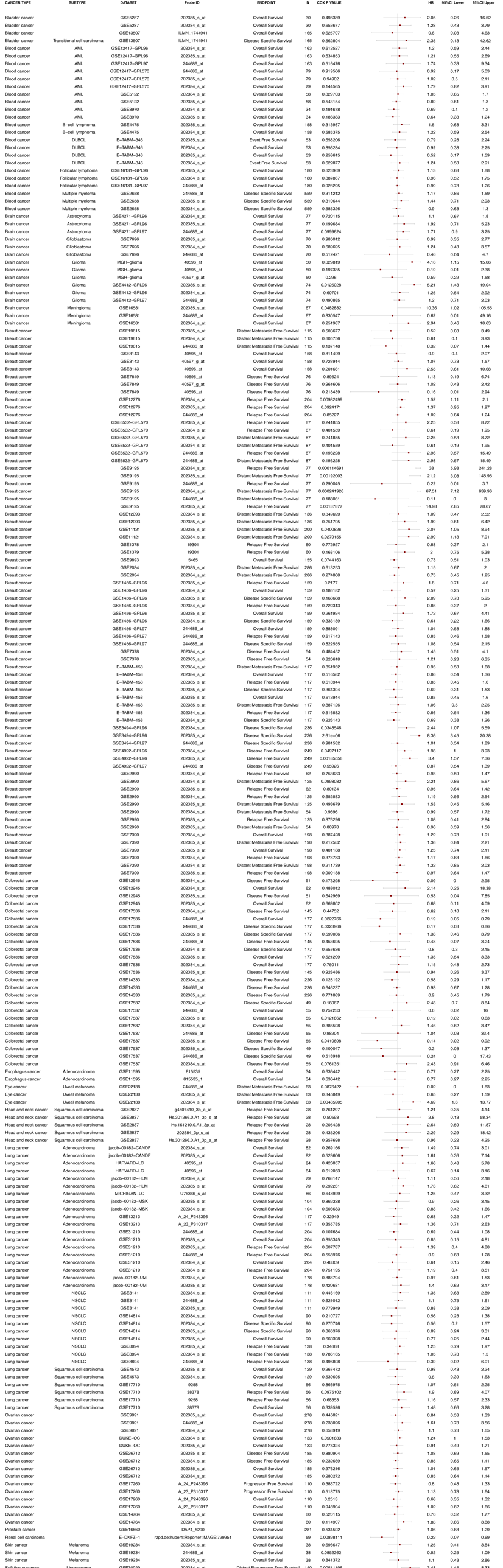

Supplement: Supplementary Figure 2 [file aging-14-203852-s002.pdf]

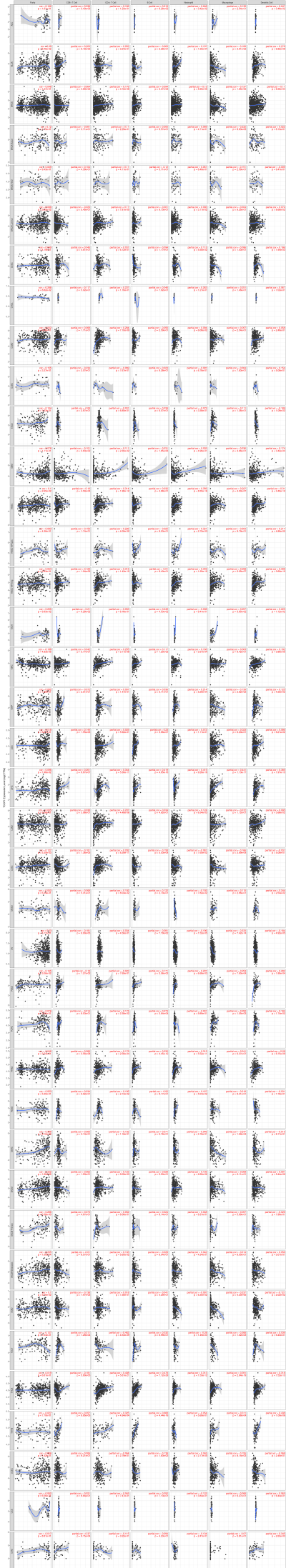

Supplement: Supplementary Figure 4 [file aging-14-203852-s003.pdf]
