# Supplementary material for: The oncogenic role of treacle ribosome biogenesis factor 1 (TCOF1) in human tumors: a pan-cancer analysis
Source: Aging (Albany NY). 2022 Jan 30;14(2):943–60. doi: 10.18632/aging.203852 (PMC8833134; doi:10.18632/aging.203852)
Supplement: Supplementary Table 3 [file aging-14-203852-s006.pdf]

## SUPPLEMENTARY TABLE

**Supplementary Table 3. Comparison of the expression of *TCOF1* between immunotherapy responders and non-responders.**

| No | PMID | Cancer type | Group | Drug | # Res | # NRes | Log2 (Fold Change) | *P* value |
|---|---|---|---|---|---|---|---|---|
| 1 | 26997480 | Melanoma | all | Anti-PD-1 (pembrolizumab and nivolumab) | 14 | 12 | 0.03 | 0.921 |
| 2 | 26997480 | Melanoma | MAPKi | Anti-PD-1 (pembrolizumab and nivolumab) | 6 | 5 | 0.196 | 0.908 |
| 3 | 26997480 | Melanoma | non-MAPKi | Anti-PD-1 (pembrolizumab and nivolumab) | 8 | 7 | –0.101 | 0.943 |
| 4 | 28552987 | Urothelial cancer | all | Anti-PD-L1 (atezolizumab) | 9 | 16 | 0.142 | 0.605 |
| 5 | 28552987 | Urothelial cancer | smoking | Anti-PD-L1 (atezolizumab) | 5 | 9 | 0.088 | 0.972 |
| 6 | 28552987 | Urothelial cancer | non-smoking | Anti-PD-L1 (atezolizumab) | 4 | 7 | 0.215 | 0.948 |
| 7 | 29033130 | Melanoma | all | Anti-PD-1 (nivolumab) | 26 | 23 | 0.31 | 0.384 |
| 8 | 29033130 | Melanoma | NIV3-PROG | Anti-PD-1 (nivolumab) | 15 | 11 | 0.103 | 0.947 |
| 9 | 29033130 | Melanoma | NIV3-NAIVE | Anti-PD-1 (nivolumab) | 11 | 12 | 0.568 | 0.739 |
| 10 | 29301960 | Clear cell renal cell carcinoma (ccRCC) | all | Anti-PD-1 (nivolumab) | 4 | 8 | 0.451 | 0.714 |
| 11 | 29301960 | Clear cell renal cell carcinoma (ccRCC) | VEGFRi | Anti-PD-1 (nivolumab) | 2 | 0 | 0 | 1 |
| 12 | 29301960 | Clear cell renal cell carcinoma (ccRCC) | non-VEGFRi | Anti-PD-1 (nivolumab) | 2 | 8 | 0.645 | 0.722 |
| 13 | 29443960 | Urothelial cancer | all | Anti-PD-L1 (atezolizumab) | 68 | 230 | –0.015 | 0.829 |
